# Four-Week Supplementation of Water-Soluble Tomato Extract Attenuates Platelet Function in Chinese Healthy Middle-Aged and Older Individuals: A Randomized, Double-Blinded, and Crossover Clinical Trial

**DOI:** 10.3389/fnut.2022.891241

**Published:** 2022-06-01

**Authors:** Zezhong Tian, Die Fan, Kongyao Li, Dan Zhao, Ying Liang, Qiuhua Ji, Xiaoli Gao, Xilin Ma, Yimin Zhao, Yuheng Mao, Huicui Meng, Yan Yang

**Affiliations:** ^1^School of Public Health (Shenzhen), Shenzhen Campus of Sun Yat-sen University, Sun Yat-sen University, Shenzhen, China; ^2^Guangdong Provincial Key Laboratory of Food, Nutrition and Health, Guangzhou, China; ^3^Guangdong Engineering Technology Center of Nutrition Transformation, Guangzhou, China; ^4^Department of Clinical Nutrition, The General Hospital of Western Theater Command, Chengdu, China; ^5^Shenzhen Nanshan Center for Chronic Disease Control, Shenzhen, China; ^6^The Eighth Affiliated Hospital, Sun Yat-sen University, Shenzhen, China

**Keywords:** water-soluble tomato extract, randomized clinical trial, platelet, Chinese healthy middle-aged and older individuals, crossover study

## Abstract

**Background and Aims:**

Platelets are linked to atherosclerotic development and pathological thrombosis. Single dose of water-soluble tomato extract (WTE) which is a natural extraction can exert anti-platelet effects after 3 or 7 h in British healthy people. However, the effects of WTE supplementation on platelet function in Chinese healthy middle-aged and older individuals have not been studied, and the effects or safety of 4-week WTE supplementation also remain unclear. The present study aims to determine the effects of WTE on platelet function, and explore the safety of 4-week WTE supplementation in Chinese healthy middle-aged and older individuals.

**Methods:**

A randomized, double-blinded, and crossover clinical trial was conducted. Firstly, 105 individuals were randomly divided into two groups that received WTE (150 mg/day) or placebo for 4 weeks. Then, after a washout period of 2 weeks, two groups exchanged groups and continued for another 4-week intervention. Platelet aggregation, P-selectin, activated GPIIbIIIa, plasma platelet factor 4 (PF4), β-thromboglobulin (β-TG), and thromboxane B_2_ (TXB_2_) were tested at baseline, 4, 6, and 10 weeks.

**Results:**

Compared with the placebo group, 150 mg/day WTE supplement for 4 weeks significantly reduced ADP-induced or collagen-induced platelet aggregation (−10.8 ± 1.8 or −3.9 ± 1.5%, *P* < 0.05), ADP-induced or collagen-induced platelet P-selectin expression (−6.9 ± 1.5 or −6.6 ± 1.3%, *P* < 0.05), ADP-induced or collagen-induced activated GPIIbIIIa (−6.2 ± 2.0 or −3.8 ± 2.0%, *P* < 0.05). Besides, 4-week intervention of 150 mg WTE per day also resulted in significant reductions in plasma PF4 (−120.6 ± 33.2 ng/mL, *P* < 0.05) and β-TG (−129.7 ± 27.5 ng/mL, *P* < 0.05) and TXB_2_ (−42.0 ± 4.0 ng/mL, *P* < 0.05), while had no effects on coagulation function and liver or renal function. Interestingly, 2-week washout period is enough to reverse the inhibitory effect of 4-week WTE supplementation on platelet function.

**Conclusion:**

WTE supplementation for 4 weeks could moderately reduce platelet activation, aggregation, and granule secretion in Chinese healthy middle-aged and older individuals, and these effects are safe. After 2-week washout period, the inhibitory effect of 4-week WTE on platelet function can be eliminated.

**Clinical Trial Registration:**

[http://www.chictr.org.cn/], identifier [ChiCTR-POR-17012927].

## Introduction

Cardiovascular diseases (CVDs), the major non-communicable diseases, are the leading cause of deaths globally (1), especially accounting for more than 40% of deaths in China (2). Global Burden of Disease (GBD) study has shown that there were an approximated 93.8 million prevalent cases of CVDs overall during 2016 in China, more than twice that of 1990, thus resulting the serious burden of CVDs in China (3).

Pathological mechanisms of CVDs are mainly atherosclerosis (AS) and thrombosis (4). A large number of studies have shown that platelets play an important role in the initiation and progression of atherothrombosis as platelet activation, aggregation and the release of platelet granule contents participated in the formation of thrombus and atherosclerotic plaques (5). A previous study has shown that the progressive decline of platelet health was observed in the middle-aged and older individuals, marked by an increase in oxidative stress and platelet hyper-activation (6). Therefore, primary prevention of CVDs by antiplatelet strategy is of considerable importance in Chinese middle-aged and older individuals.

At present, various anti-platelet drugs (such as aspirin, clopidogrel, and ticagrelor) are widely used for prevention and treatment of CVDs such as atherosclerosis (7, 8). However, a large number of reports indicated that large-scale use of these drugs may increase bleeding risk or cause thrombocytopenia (9, 10). These side effects limit the application of anti-platelet drugs in the early prevention and treatment of thrombotic diseases. Therefore, other safe and effective anti-platelet strategies for early prevention should be developed to reduce the risks of CVDs in middle-aged and older individuals.

Epidemiological studies have found that there was a negative association between tomato consumption and the incidence of CVDs in Mediterranean countries (11, 12). However, some studies have shown that the relationship between lycopene rich in tomatoes and CVDs is not strong (11, 13), thus suggesting that other unidentified compounds in tomatoes may play a role in reducing the risk of CVDs. Further *in vitro* studies showed that water-soluble tomato extract (WTE) exerted inhibitory effect on ADP-induced platelet activation and ADP- or collagen-induced platelet aggregation (14). WTE is a natural mixture that is obtained from fresh tomatoes (*Lycopersicon esculentum*) by physically removing fat-soluble components in tomatoes, and mainly composed by adenosine, chlorogenic acid, and rutin (14). Previous acute clinical trials have shown that a single dose of WTE inhibited platelet aggregation and activation after 3 or 7 h in the British adults (14, 15). Recent reviews have shown that WTE was a potential therapeutic agent for inhibiting platelet function and might circumvent the negative drawbacks of using aspirin, clopidogrel, or ticagrelor (16, 17). However, the effect and safety of 4-week administration of WTE remains unclear, and there is no study exploring the effect of WTE on platelet function in Chinese middle-aged and older individuals. In view of differences in response of platelet to anti-platelet drugs among different races (18, 19), the lack of study in other races limits the popularization of WTE to a wider population in other countries.

To address these issues, our present study aimed to conduct a randomized placebo-controlled crossover trial to determine the effect and safety of 4-week administration of WTE on platelet function in Chinese middle-aged and older individuals.

## Materials and Methods

### Study Population

All the subjects were recruited from the medical examination center of the first affiliated hospital of Sun Yat-sen University and community health center through flyers, medical record reviews, or clinicians’ recommendations in Guangzhou, Guangdong, China. This study was approved by the Ethics Committee of Sun Yat-sen University (No. 2016036), and was conducted in strict accordance with the principles of the Declaration of Helsinki. This trial was registered at Chinese clinical trial registry as ChiCTR-POR-17012927. All participants signed written informed consent forms prior to enrollment.

The inclusion criteria were: (1) men or women aged 40–65 years; (2) without serious vascular or hematological diseases; and (3) normal laboratory tests for hematuria, liver and kidney function, blood glucose, and lipids. The exclusion criteria were: (1) with the history of hypertension, infectious disease, hemostatic disorders, diabetes mellitus, and CVDs; (2) agents use that could influence platelet function; (3) lactating or pregnant women; and (4) allergic to tomatoes and their ingredients.

### Study Design

**FIGURE 1:**
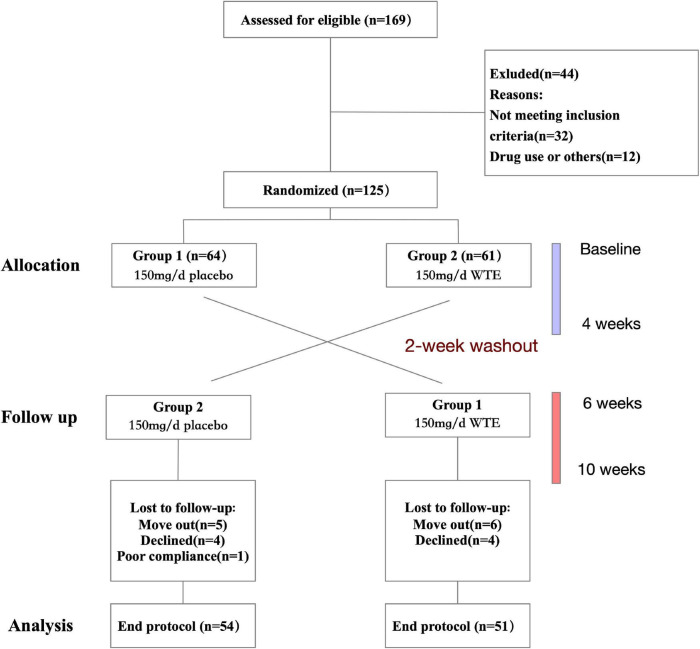
Flow diagram of the study population.

This was a randomized crossover-designed controlled trial. First, a total of 105 eligible subjects were stratified by gender and then randomly divided into two group 1 or group 2 by random numbers generated by SPSS v22.0 (SPSS Inc., Chicago, IL, United States). Referring to other previous randomized controlled trials which focused on the effects of 4-week supplementation for other nutritional supplements rich in phytochemical (20–23), we took 4 weeks as the duration of WTE supplementation in this study. Individuals in group 1 took one WTE tablet (150 mg/day) and individuals in group 2 take one placebo tablet daily for 4 weeks, followed by a 2-week washout period. Then, the two groups switched groups and continued to take WTE (150 mg/day) or placebo tablets for another 4 weeks (Figure 1). During the 10-week trial period, all subjects were instructed to maintain their usual diet and lifestyle, but to refrain from tomatoes and tomato products. The 24-h dietary recall data were recorded on 3 consecutive days, and International Physical Activity Questionnaire (IPAQ) scores were collected *via* face-to-face interviews at baseline and after intervention (24, 25).

A technician who did not participate in the experiments, data collection or analysis was responsible for randomization, and the management of the packaged supplements. Participants, investigators, and laboratory technicians were blinded to the treatment assignments.

Use PASS software (version 11.0, NCSS Inc.) to estimate the sample size required for the study based on the conventional assumption of a two-tailed α level of 0.05 and β level of 0.10. According to the previous study (15), the change rates of platelet aggregation in the intervention group and the control group were −9.7 and −3.1% after the intervention of WTE. Based on a two-tailed α level of 0.05 and β level of 0.10, 42 participants in each group were needed. Considering a 10% loss to follow-up rate, 47 subjects are needed in each group at least.

### Study Supplements

Water-soluble tomato extract tablets and placebo tablets were provided by DSM (Netherlands) and By-health (China). The WTE tablets contain 150 mg WTE (Fruitflow) which comprises nucleosides, polyphenols, and flavonoids purified from fresh tomatoes (*L. esculentum*) by using solid-phase extraction, as described in previous study (14) (refer to Supplementary Table 1 for the ingredients of the WTE tablets). The placebo tablets contained only maltodextrin. The weight, appearance, taste, and packaging of WTE tablets and placebo tablets were same.

### Anthropometric Analyses and Assessment of Biochemical Biomarkers

Body weight, height, waist circumference (WC), hip circumference (HC), heart rate (HR), and blood pressure (BP) were measured according to standard protocols, as previously described (24, 25).

Fasting blood samples were obtained at 8–9 a.m. from each subject’s antecubital vein with a 21-gauge needle at the beginning and at weeks 4, 6, and 10 of the trial. Whole human blood was collected into a Vacutainer tube containing 3.8% sodium citrate (1/9, v/v) *via* venipuncture. The blood sample was then centrifuged at 1,700 × *g* for 15 min at 4°C to obtain plasma, and the blood sample with sodium citrate was then centrifuged at 200 × *g* for 10 min at 22°C to obtain platelet-rich plasma (PRP) (26). PRP was immediately used to assay platelet aggregation and activation or other related parameters. Plasma samples were stored at −80°C until subsequent analyses.

Fasting blood samples were subjected to LDL-C, HDL-C, serum TG, serum TC, fasting blood glucose (FBG), urea, uric acid (UA), creatinine, alanine aminotransferase, total protein, albumin, and globulin. Lipid profile analyses were performed on the Cobas c311 automated assay analyzer (c311, Roche Diagnostics, Switzerland). Enzymatic methods were used to determine the concentrations of HDL-C, LDL-C, TC, and TG. Other biochemical analyses included FBG, urea, UA, creatinine, alanine aminotransferase, total protein, albumin, and globulin were measured using the Cobas c311 automated assay analyzer. Prothrombin time (PT), activated partial thromboplastin time (APTT), thrombin clotting time (TT), and plasma fibrinogen (Fib) estimations were performed on a Sysmex5100 system (Siemens Healthineers, Malvern, PA, United States).

The platelet granule contents including platelet factor 4 (PF4), β-thromboglobulin (β-TG), and thromboxane B2 (TXB_2_) in plasma were tested using an enzyme linked immunosorbent assay (ELISA) kit according to the manufacturer’s instructions. The PF4 and β-TG in plasma were detected *via* the double antibody sandwich method of ELISA (Abcam, United Kingdom). TXB_2_ is the stable product of the non-enzymatic hydration of thromboxane A2 (TXA_2_), thus the production of TXA_2_ *in vivo* is typically monitored by measurement of TXB_2_ (27). Therefore, as previous studies (28–31), we chose the R&D’s TXB_2_ competitive ELISA kit (R&D Systems, United States) to detect TXB_2_ for reflecting the production of TXA_2_. The SPARK^®^ multimode microplate reader (Tecan, Switzerland) was used to record ODs at 450 nm.

### Detection of Platelet Surface CD62P and GPIIbIIIa Expression by Flow Cytometric Analysis

In the clinical trial, PRP (5 × 10^6^ platelets/mL) were labeled with FITC-conjugated anti-human CD62P antibody or PAC-1 antibody for 20 min and followed the stimulation of ADP or collagen, samples were then fixed with 1% paraformaldehyde and analyzed using a CytoFLEX flow cytometer (Beckman Coulter, CA, United States), and data were analyzed with CytExpert 2.0 (Beckman Coulter, CA, United States), as previously described (32, 33).

### Detection of Platelet Aggregation by Light Aggregometry

Briefly, 500 μL of fresh PRP from citrated blood was incubated in a transparent cuvette at 37°C for 5 min. Then the cuvette was transferred into the detection hole of Chronolog aggregometer (Chrono-Log Corp., PA, United States) and stimulated by 5 μmol/L ADP or 2 μg/mL collagen. At least 500 μL of PRP need to be used for platelet aggregation test at a time, which means that a lot of whole blood needs to be collected. This is not in line with the principles of ethics. In addition, previous acute studies in Britain have found that WTE significantly inhibited collagen- and ADP-induced platelet aggregation (14, 15). Therefore, we measured platelet aggregation stimulated by collagen and ADP. Platelet aggregation was evaluated on a Chronolog aggregometer in PRP (3.0 × 10^8^ platelets/mL) at 37°C with a sample stir speed of 1,000 rpm, and the maximum-reversible platelet aggregation was monitored and recorded for at least 6 min, as previously described (33, 34).

### Statistical Analysis

SPSS 22.0 software was used for statistical analysis. In the clinical study, the data are expressed as the mean ± standard error of the mean (SEM) unless otherwise stated. For categorical variables, analysis was performed using the chi-square test. The changes after 4-week WTE supplementation or placebo supplementation were calculated as values after 4-week intervention (at 4 or 10 weeks) deducted from the values at baseline (at 0 week or after 2-week washout period). For continuous variables, variables were checked for normal distribution and were assessed by Kruskal–Wallis test if they were not normally distributed. The comparability of the two groups at baseline were assessed by one-way analysis of variance (ANOVA).

The effects of treatments were evaluated using one-way ANOVA, comparisons of each index at baseline (at 0 week or after 2-week washout period) and after 4-week intervention (at 4 or 10 weeks) between two groups were conducted using the Student’s *t*-test for unpaired data. The mean changes of each index between two groups after 4-weeks intervention were compared using the Student’s *t*-test for unpaired data. To explore the reversibility of 4-week supplementation of WTE, one-way ANOVA were used to assess changes between two groups at baseline, 4, 6, and 10 weeks. Stratified analyses were performed with ANOVA to compare the changes of platelet function after 4-week treatment stratified by gender. Differences were considered statistically significant at *P* < 0.05.

### Role of Funding Source

All funders of the present study (namely National Natural Science Foundation of China, Shenzhen Science, Technology, and Innovation Commission, By-health Research Foundation and Scientific Research Foundation of Chinese Nutrition Society) only provided financial support and did not participate in the design, data collection, data analysis, interpretation of the clinical trial, and the writing of this manuscript.

## Results

### Characteristics and Diet Monitoring

A total of 105 individuals completed the trial and were included in the analysis (Figure 1). The characteristics of 105 individuals are shown in Table 1. At baseline, 105 individuals were comparable in terms of age, sex, height, BMI, WC, HC, HR, SBP, DBP, lipid profile, and routine blood test (Table 1 and Supplementary Table 2). After intervention, there was no significant difference between two groups in terms mentioned above (Table 1 and Supplementary Table 2). In addition, there was no significant difference between two groups in daily physical activity status or the intake of energy, total protein, carbohydrates, lipids, dietary fiber, and vitamins at baseline and after intervention (Supplementary Table 3).

**TABLE 1:** Anthropometrics of all participants at baseline and after treatment.

	Group 1 (*n* = 54)		Group 2 (*n* = 51)	
	Baseline	After intervention	Baseline	After intervention
Gender (M/F)	16/38		15/36	
Age, years	54.0 ± 7.4		53.6 ± 9.7	
Weight (kg)	63.5 ± 11.5	63.4 ± 11.3	63.6 ± 11.5	63.6 ± 11.5
Height (cm)	160.3 ± 7.8	160.4 ± 7.8	160.3 ± 7.8	160.3 ± 7.8
BMI (kg/m^2^)	24.6 ± 3.2	24.6 ± 3.2	24.6 ± 3.2	24.6 ± 3.3
WC (cm)	84.746 ± 11.5	85.8 ± 8.5	84.4 ± 11.2	85.6 ± 10.6
HC (cm)	96.4 ± 7.0	95.9 ± 7.2	96.11 ± 6.8	96.4 ± 6.9
SBP (mmHg)	118.2 ± 16.2	118.8 ± 14.6	116.9 ± 14.6	117.9 ± 15.0
DBP (mmHg)	78.1 ± 10.6	79.1 ± 10.6	77.2 ± 10.0	80.2 ± 19.5
HR (beats/min)	73.4 ± 9.0	72.6 ± 8.0	73.7 ± 8.4	73.3 ± 8.3
ALT (U/L)	20.8 ± 10.8	22.4 ± 13.8	22.3 ± 15.7	20.4 ± 12.9
Total protein (g/L)	74.4 ± 3.4	72.8 ± 3.6	74.0 ± 5.7	73.0 ± 3.9
Albumin (g/L)	46.0 ± 2.5	45.6 ± 2.6	45.9 ± 4.1	46.3 ± 2.6
Globulin (g/L)	28.3 ± 3.0	27.2 ± 3.6	28.3 ± 3.1	26.6 ± 4.1
A/G	1.6 ± 0.2	1.7 ± 0.3	1.7 ± 0.2	1.8 ± 0.6
Urea (mmol/L)	4.9 ± 1.1	5.1 ± 1.4	4.8 ± 1.3	4.8 ± 1.1
Creatinine (μmol/L)	74.1 ± 17.6	73.1 ± 17.8	74.4 ± 15.3	72.6 ± 15.6
UA (μmol/L)	359.2 ± 100.4	352.4 ± 91.6	385.5 ± 104.8	346.3 ± 79.1
TG (mmol/L)	1.6 ± 0.9	1.4 ± 0.8	1.8 ± 1.1	1.7 ± 1.2
TC (mmol/L)	5.1 ± 1.0	5.1 ± 1.1	5.2 ± 0.9	5.2 ± 0.9
HDL-c (mmol/L)	1.4 ± 0.6	1.3 ± 0.3	1.4 ± 0.4	1.4 ± 0.5
LDL-c (mmol/L)	3.3 ± 0.9	3.4 ± 1.0	3.4 ± 0.8	3.5 ± 0.8

*The data are expressed as mean ± SEM. There were no significant differences for any variable between the two groups at the baseline or after intervention. M, male; F, female; BMI, body mass index; NC, neck circumference; WC, waist circumference; HC, hip circumference; SBP, systolic blood pressure; DBP, diastolic blood pressure; HR, heart rate; TC, total cholesterol; HDL-c, high density lipoprotein cholesterol; LDL-c, low density lipoprotein cholesterol; TG, total triglyceride; UA, uric acid; ALT, alanine aminotransferase; A/G, albumin/globulin.*

### Water-Soluble Tomato Extract Supplementation Inhibited Platelet Activation and Aggregation in Chinese Healthy Middle-Aged and Older Individuals

At baseline, there were no significant differences in platelet activation and aggregation between two groups. Subjects consumed 150 mg of WTE or placebo tablet daily for 4 weeks. After 4-week intervention, 150 mg/day of WTE significantly reduced the percentage of ADP- or collagen-induced activated GPIIbIIIa positive platelets, with baseline levels of 51.8 ± 1.4 or 21.3 ± 1.5% and post-intervention levels of 45.6 ± 1.8 or 17.5 ± 1.2%, respectively (*P* < 0.05, Table 2). Four weeks of 150 mg/day WTE also significantly decreased the percentage of ADP- or collagen-induced P-selectin positive platelets, with baseline values of 51.2 ± 1.0 or 32.3 ± 1.0% and post-intervention values of 44.3 ± 1.1 or 25.7 ± 0.9% (*P* < 0.05, Table 2). Similarly, 150 mg/day WTE resulted in significant reductions in platelet aggregation stimulated by ADP or collagen, with baseline values of 72.3 ± 1.4 and 81.1 ± 1.2% and post-intervention values of 61.5 ± 1.6 and 77.2 ± 0.9% after 4-week supplementation (*P* < 0.05, Table 2). After 4-week intervention, placebo did not significantly change platelet activation and aggregation in Chinese healthy middle-aged and older individuals (*P* > 0.05, Table 2). In addition, there were significant differences between 150 mg/day WTE group and the placebo group in terms of platelet activation and aggregation after 4-week intervention (*P* < 0.05, Table 2).

**TABLE 2:** Effects of WTE on platelet activation and aggregation in healthy middle-aged and older individuals.

	Placebo (*n* = 105)			WTE (*n* = 105)			
	Baseline	After intervention	Mean change^a^	Baseline	After intervention	Mean change^a^	*P*-value^e^
ADP-induced platelet aggregation (%)	71.6 ± 1.5	70.0 ± 1.8	−1.6 ± 2.1	72.3 ± 1.4	61.5 ± 1.6^b, c^	−10.8 ± 1.8^d^	<0.001
Collagen-induced platelet aggregation (%)	80.4 ± 0.9	81.9 ± 0.9	1.5 ± 1.1	81.1 ± 1.2	77.2 ± 0.9^b, c^	−3.9 ± 1.5^d^	0.004
ADP-induced platelet P-selectin (%)	48.5 ± 1.0	53.4 ± 1.3	4.8 ± 1.4	51.2 ± 1.0	44.3 ± 1.1^b, c^	−6.9 ± 1.5^d^	<0.001
Collagen-induced platelet P-selectin (%)	31.0 ± 0.9	30.2 ± 1.0	−0.8 ± 1.2	32.3 ± 1.0	25.7 ± 0.9^b, c^	−6.6 ± 1.3^d^	<0.001
ADP-induced activated platelet GPIIbIIIa (%)	50.1 ± 1.5	55.2 ± 1.8	5.1 ± 1.7	51.8 ± 1.4	45.6 ± 1.8^b, c^	−6.2 ± 2.0^d^	0.001
Collagen-induced activated platelet GPIIbIIIa (%)	21.7 ± 1.5	24.7 ± 1.2	3.0 ± 1.9	21.3 ± 1.5	17.5 ± 1.2^b, c^	−3.8 ± 2.0^d^	0.003

*The data are expressed as mean ± SEM. At the baseline, there were no significant differences for any variable between the two groups. In the placebo group, there are no significant differences for any variable after the 4-weeks treatment.*

*^a^Calculated as value at 4 weeks – value at baseline.*

*^b^P < 0.05 vs 4 weeks in the placebo group, assessed by unpaired Student’s t-test.*

*^c^P < 0.05 vs baseline in the WTE group, assessed by paired Student’s t-test.*

*^d^P < 0.05 vs mean changes in the placebo group, assessed by unpaired Student’s t-test.*

*^e^Effects of the treatment on these variables were evaluated by one-way ANOVA.*

The percentage of ADP-induced activated GPIIbIIIa positive platelets from baseline to 4 weeks significantly decreased in the 150 mg/day WTE (−6.2 ± 2.0%, *P* < 0.05, Table 2). The percentage of collagen-induced activated GPIIbIIIa positive platelets from baseline to 4 weeks also significantly decreased in the 150 mg/day WTE groups (−3.8 ± 2.0%, *P* < 0.05), compared with the placebo group (3.0 ± 1.9%, Table 2). Similarly, the change in ADP- or collagen-induced platelet aggregation from baseline to 4 weeks in the 150 mg/day WTE group (−10.8 ± 1.8 or −3.9 ± 1.5%) was significantly different from the change in the placebo group (−1.6 ± 2.1 or 1.5 ± 1.1%, *P* < 0.05) (Table 2). Besides, there were significant differences between 150 mg/day WTE group (−6.9 ± 1.5 or −6.6 ± 1.3%) and the placebo group (4.8 ± 1.4 or −0.8 ± 1.2%) in the percentage of ADP- or collagen-induced P-selectin positive platelets (*P* < 0.05, Table 2). Stratified analyses indicated that WTE could significantly inhibit platelet activation and aggregation in whether male or female, and WTE might have more robust inhibition on platelet activation and aggregation among females (Supplementary Tables 4, 5).

### Water-Soluble Tomato Extract Supplementation Reduced the Level of Platelet Granule Secretion in Chinese Healthy Middle-Aged and Older Individuals

One hundred and five individuals were comparable between two groups in terms of platelet granule secretion at baseline (*P* > 0.05, Table 3). Compared with baseline values, the 4 weeks of placebo supplementation did not significantly alter any of the variables (*P* > 0.05, Table 3). However, a 4-week intervention of 150 mg WTE per day can significantly reduce plasma β-TG from baseline value of 495.8 ± 19.1 ng/mL to post-intervention value of 366.1 ± 19.2 ng/mL (*P* < 0.05, Table 3). Four weeks of WTE at 150 mg/day also significantly decreased plasma PF4, with baseline value of 397.3 ± 24.5 ng/mL and post-intervention value of 276.7 ± 27.1 ng/mL (*P* < 0.05, Table 3). Similarly, 150 mg/day WTE resulted in significant reductions in TXB_2_, with baseline value of 160.5 ± 4.5 ng/mL and post-intervention value of 118.5 ± 6.8 ng/mL after 4-week supplementation (*P* < 0.05, Table 3).

**TABLE 3:** Effects of WTE on platelet granule secretion in healthy middle-aged and older individuals.

	Placebo (*n* = 105)			WTE (*n* = 105)			
	Baseline	After intervention	Mean change^a^	Baseline	After intervention	Mean change^a^	*P*-value^e^
β-TG (ng/mL)	519.4 ± 17.9	456.9 ± 17.9	−62.4 ± 24.6	495.8 ± 19.1	366.1 ± 19.2^b, c^	−129.7 ± 27.5	<0.001
PF4 (ng/mL)	393.0 ± 29.6	430.0 ± 27.4	37.0 ± 40.3	397.3 ± 24.5	276.7 ± 27.1^b, c^	−120.6 ± 33.2^d^	<0.001
TXB_2_ (ng/mL)	165.8 ± 4.0	164.8 ± 4.6	−1.0 ± 2.4	160.5 ± 4.5	118.5 ± 6.8^b, c^	−42.0 ± 4.0^d^	<0.001

*The data are expressed as mean ± SEM. At the baseline, there were no significant differences for any variable between the two groups. In the placebo group, there are no significant differences for any variable after the 4-weeks treatment.*

*^a^Calculated as value at 4 weeks – value at baseline.*

*^b^P < 0.05 vs 4 weeks in the placebo group, assessed by unpaired Student’s t-test.*

*^c^P < 0.05 vs baseline in the WTE group, assessed by paired Student’s t-test.*

*^d^P < 0.05 vs Mean changes in the placebo group, assessed by unpaired Student’s t-test.*

*^e^Effects of the treatment on these variables were evaluated by one-way ANOVA.*

After 4-week intervention, there were significant differences between 150 mg/day WTE group and the placebo group plasma β-TG, PF4 and TXB_2_ (*P* < 0.05, Table 3). The plasma β-TG from baseline to 4 weeks significantly decreased (−129.7 ± 27.5 ng/mL) in the 150 mg/day WTE group (Table 3). The decrease in plasma PF4 from baseline to 4 weeks in the 150 mg/day WTE group (−120.6 ± 33.2 ng/mL) was significantly different from the change in the placebo group (37.0 ± 40.3 ng/mL, *P* < 0.05), as shown in Table 3. In addition, compared with the placebo group (−1.0 ± 2.4 ng/mL), the level of plasma TXB_2_ from baseline to 4 weeks was significantly reduced in the 150 mg/day WTE group (−42.0 ± 4.0 ng/mL, *P* < 0.05), as shown in Table 3. Stratified analyses indicated that WTE could significantly inhibit platelet granule secretion in whether male or female, and WTE might have more robust inhibition on platelet granule secretion among females (Supplementary Tables 4, 5).

### The Inhibitory Effect of Water-Soluble Tomato Extract on Platelet Function Can Be Eliminated After 2-Week Washout Period

After 4-week intervention, 150 mg/day WTE can significantly reduce platelet activation, aggregation, and granule contents, compared with placebo (*P* < 0.05, Tables 2, 3). To evaluate the reversibility of anti-platelet effect of WTE, we further performed a 2-week washout period for all volunteers after 4-week intervention. Interestingly, after a 2-week washout period, there was no significant difference in the percentage of ADP- or collagen-induced activated GPIIbIIIa positive platelets, the percentage of ADP- or collagen-induced P-selectin positive platelets, platelet aggregation stimulated by ADP or collagen, plasma β-TG and TXB_2_ between 150 mg/day WTE group and placebo group (*P* > 0.05, Supplementary Tables 6, 7). These results showed that effects of 4-week WTE supplement on platelet activation, aggregation, and granule contents are reversible.

### Safety Evaluation

Considering the anti-platelet ability of WTE, we carefully determined whether the clotting pathways were affected by WTE alongside anti-platelet effects. We therefore examined the coagulation function of subjects and found that 4-week intervention of WTE had no any adverse effects on PT, PT-INR, PT-R, APTT, TT, and Fib in Chinese healthy middle-aged and older individuals (*P* > 0.05, Table 4). Similarly, there was also no significant difference between two groups in urea, UA, creatinine, alanine aminotransferase, total protein, albumin, and globulin at baseline and after intervention, and these results suggested that 4-week intervention of WTE had no significant effect on liver and kidney function in Chinese healthy middle-aged and older individuals (*P* > 0.05, Table 1). In addition, no adverse events were reported during the intervention period.

**TABLE 4:** Effects of WTE on coagulation function in healthy middle-aged and older individuals.

	Placebo (*n* = 105)		WTE (*n* = 105)	
	Baseline	After intervention	Baseline	After intervention
PT (s)	12.0 ± 0.6	11.0 ± 0.8	10.8 ± 0.8	10.9 ± 0.7
PT-INR	0.9 ± 0.07	0.9 ± 0.07	0.9 ± 0.07	0.9 ± 0.06
PT-R	0.9 ± 0.06	0.9 ± 0.06	0.9 ± 0.06	0.9 ± 0.06
APTT (s)	30.0 ± 3.7	29.3 ± 4.0	29.7 ± 3.8	29.3 ± 3.6
TT (s)	18.6 ± 1.2	18.2 ± 1.3	18.4 ± 1.2	18.2 ± 1.2
Fib (g/L)	2.8 ± 0.6	2.9 ± 0.6	2.8 ± 0.6	2.8 ± 0.6

*The data are expressed as mean ± SEM. At the baseline, there were no significant differences for any variable between the two groups. There are no significant differences for any variable between two groups after the 4-weeks intervention. PT, prothrombin time; PT-INR, prothrombin time-international normalized ratio; PT-R, prothrombin time-ratio; APTT, activated partial thromboplasting time; TT, thrombin time; Fib, fibrinogen.*

## Discussion

In the present study, we set out to investigate the effects of WTE on platelet function in Chinese healthy middle-aged and older adults. For this purpose, we sought to perform a crossover comparison of 150 mg WTE and placebo, in Chinese healthy middle-aged and older adults fitting the target population for WTE consumers. The results have shown that supplementation of WTE can reduce plasma PF4, β-TG, TXA2, and inhibited agonist-induced platelet activation and aggregation in Chinese healthy middle-aged and older adults, and the effects of 4-week WTE supplementation on platelet activation, aggregation, and granule contents are reversible after the 2-week washout period.

Platelets can be activated first and then aggregate after the stimulation of ADP and Collagen (37). PF4 and β-TG are platelet-specific granule contents, which are lower in the platelet resting state, and can multiply when platelets are stimulated, and therefore can be widely used as indicators reflecting the release state of platelet granule secretion (38). Controlling platelet hyperactivity has become one of the important strategies to prevent the development of atherothrombosis and CVDs. Our previous experiments have indicated that WTE directly inhibited platelet activation, aggregation and thrombosis by using gel-filtered platelets and FeCl_3_-induced mesenteric artery thrombosis model (39). We conducted a randomized controlled crossover clinical trial to explore whether WTE can also reduce platelet activation and aggregation, and its effect on platelet granule secretion. WTE is now authorized by the European Food Safety Authority for daily consumption with 150 mg in the format of powder, tablet, or capsule (35). Therefore, we used the doses of 150 mg/day for our clinical trial. Our present studies proved that WTE supplementation significantly inhibited ADP- or collagen-induced platelet aggregation, platelet surface P-selectin expression, GPIIbIIIa activation, and TXA_2_ generation in Chinese healthy middle-aged and older individuals. Meanwhile, WTE supplementation can significantly reduce the level of platelet granule secretion including plasma PF4 and β-TG. These are consistent with previous studies in European (15, 36), suggesting that the anti-platelet properties of WTE is applicable not only to European healthy individuals, but also to Chinese healthy middle-aged and older individuals.

Clinical trials focused on nutrients have shown that the duration of nutrient supplementation is an important factor affecting its improvement effect (24, 25, 40–43). Previous studies explore that WTE supplementation exerts anti-platelet effects after 3 or 7 h, which is relatively short (15, 36). Niamh O’Kennedy etc., has found that WTE resulted in significant reductions about −4.5% in platelet aggregation stimulated by ADP after 7 h (36). Another study found that single dose of WTE could further reduce ADP-induced platelet aggregation (−11.7 ± 5.3%) combined with 7-day treatment of 75 mg aspirin (15). Our present study suggested that WTE can significantly reduce ADP-induced platelet aggregation (−10.8 ± 3.5%) after 4-week intervention, and this inhibitory effect of 4-week WTE intervention is better than the acute treatment of WTE and is similar to 7-day treatment of 75 mg aspirin combined with WTE. Besides, our study found that WTE significantly reduced collagen- or ADP-induced platelet activation and granule contents after 4-week intervention. These evidences indicated that daily intake of WTE for 4-week intervention might stably reduce platelet activation, aggregation, and granule contents in Chinese healthy middle-aged and older individuals.

Many anti-platelet drugs irreversibly inhibit platelet function and affect the life span of circulating platelets, which cause extra bleeding risks (9, 44). For instance, aspirin recognized as an irreversible inhibitor of cyclo-oxygenase is commonly used as oral anti-platelet drugs (45). Meta-analysis of 13 trials with 164,225 participants has found that the use of aspirin was not only associated with a lower risk of cardiovascular events, but also with an increased risk of major bleeding due to its irreversible inhibit on platelet (10, 45). Previous trials have proved that 150 mg of WTE for 7 h or 7 days did not affect prothrombin time and TT (15, 46). Considering that WTE possessed significant anti-platelet ability, we also carefully determined whether the clotting pathways are affected by WTE alongside anti-platelet effects in Chinese healthy middle-aged and older individuals. In our clinical trial, WTE exerts anti-platelet ability with no any adverse effects on the coagulation function related to prothrombin system after 4-week supplementation. Our previous animal experiment also found that WTE had no effect on the bleeding time of mice (39). This is consistent with our present study. Interestingly, our present study also found that the inhibitory effect of 4-week WTE on platelet function in Chinese healthy middle-aged and older individuals can be eliminated after 2-week washout period. The results indicated that the inhibitory effect of WTE on platelets was not similar with anti-platelet drugs and could be reversed after a washout period. In addition, we found that 4-week intervention of WTE had no effect on liver function and kidney function in Chinese healthy middle-aged and older individuals. This evidence indicated that daily intake of WTE is safe in Chinese healthy middle-aged and older individuals, and it has the potential to be applied for primary prevention of CVDs.

Recent *in vitro* study (47) quantitatively analyzes the effect of WTE on the inhibition of agonist-induced platelets with the involvement of several signaling molecules proteomics, and found that compared WTE-treated agonist-induced platelets with only agonist-induced platelets, 60 proteins were upregulated and 10 proteins were downregulated. Further validation indicated that WTE might also exert antiplatelet properties *via* decreasing Akt, glycogen synthase kinase 3β, p38 mitogen-activated protein kinase (MAPK), and heat shock protein (Hsp27) phosphorylation (47). Our previous *in vitro* experiments have also indicated that the inhibition of WTE on platelet activation and aggregation might be partly due to regulating the signaling pathway of PI3K/Akt and MAPKs (39). These results suggested that tomato might play an anti-platelet role *via* variety of signal pathways.

High performance liquid chromatography (HPLC) results revealed that WTE contains three main active ingredients, namely, nucleoside derivatives, phenolic conjugates, and flavonoid derivatives; the most representative among the three components are adenosine, chlorogenic acid, and rutin, respectively (35). These three substances can be used to quantify and evaluate the quality of WTE and ensure that WTE is as close to fresh tomatoes as possible. In general, nucleoside, polyphenols, and flavonoid provided platelet protection and improved platelet activation, adhesion and aggregation (48–50). Hence, the inhibitory effects of WTE on platelet granule secretion may be attributed to these three components, and we will further clarify the component which plays most important role on antiplatelet effects by non-targeted metabonomics in further research.

### Strengths and Limitations

There are some strengths and limitations in our present study. The double-blind, randomized, placebo-controlled, and crossover trial design is the major strength of this study. Different from the previous trials only focused on the effects of single-dose WTE supplementation after 3 or 7 h in Britain, our present crossover trial up to 4-week WTE supplementation can be more conducive to guiding the application of WTE in Chinese. Another strength is that we tested platelet activation and aggregation at baseline and during follow-up, which are required to be detected within two hours right after blood collection, to determine the effect and reversibility of WTE on platelet in Chinese healthy middle-aged and older individuals. To monitor their dietary habits and physical activity, we maintained their usual dietary intake and physical activities *via* 24-h dietary recall data on 3 consecutive days and an IPAQ during the trial. It may be another strength of our study. Meanwhile, some limitations also existed in the study. The findings of our study which focused on Chinese healthy middle-aged and older individuals just indicate a potential benefit for primary prevention of CVDs in Chinese healthy middle-aged and older individuals, and the primary or secondary prevention of WTE on individuals with metabolic diseases merits further clinical study. As in most other randomized controlled trials using the maximum-reversible platelet aggregation as main outcome (51–53), our present study only focused on the effect of WTE on the maximum-reversible platelet aggregation, without evaluating its effect on the maximum irreversible aggregation rate. This may be another limitation of our study.

## Conclusion

In summary, 150 mg WTE per day for 4-week supplementation can significantly inhibit platelet activation, aggregation, and granule secretion in Chinese healthy middle-aged and older individuals, and the effect of 4-week WTE is safe. After 2-week washout period, the inhibitory effect of 4-week WTE supplementation on platelet function can be eliminated. This study provides novel evidence to support the importance of WTE as a protective food supplementary for early prevention of CVDs in Chinese healthy middle-aged and older individuals.

## Data Availability Statement

Due to the principles of ethics and data privacy security, the datasets presented in this article are not readily available. After publication, deidentified participant data, described in the manuscript, study protocol, statistical analysis plan, and informed consent form will be made available upon reasonable request via email to the corresponding author.

## Ethics Statement

The studies involving human participants were reviewed and approved by the Ethics Committee of Sun Yat-sen University (No. 2016036). The patients/participants provided their written informed consent to participate in this study.

## Author Contributions

YY designed the trial. ZT, DF, KL, DZ, YL, QJ, XG, XM, YZ, and YM conducted all trial steps. ZT, DF, and KL conducted laboratory analyses. ZT and DF performed statistical analysis and drafted the manuscript. YY and HM revised and edited the draft manuscript. All authors read and approved the final manuscript.

## Conflict of Interest

The authors declare that the research was conducted in the absence of any commercial or financial relationships that could be construed as a potential conflict of interest.

## Publisher’s Note

All claims expressed in this article are solely those of the authors and do not necessarily represent those of their affiliated organizations, or those of the publisher, the editors and the reviewers. Any product that may be evaluated in this article, or claim that may be made by its manufacturer, is not guaranteed or endorsed by the publisher.

## Abbreviations

CVD: cardiovascular disease
GBD: Global Burden of Disease
WTE: water-soluble tomato extract
IPAQ: International Physical Activity Questionnaire
BW: body weight
BH: body height
NC: neck circumference
WC: waist circumference
HC: hip circumference
HR: heart rate
BP: blood pressure
PRP: platelet-rich plasma
LDL-C: low-density lipoprotein cholesterol
HDL-C: high-density lipoprotein cholesterol
TG: triglyceride
TC: total cholesterol
FBG: fasting blood glucose
UA: uric acid
PT: prothrombin time
APTT: activated partial thromboplastin time
TT: thrombin clotting time
Fib: fibrinogen
PF4: platelet factor 4
β -TG: β -thromboglobulin
TXB_2_: thromboxane B2
ANOVA: analysis of variance
SEM: standard error of the mean.

## References

[1] GBD 2016 Causes of Death Collaborators. Global, regional, and national age-sex specific mortality for 264 causes of death, 1980-2016: a systematic analysis for the global burden of disease study 2016. *Lancet.* (2017) 390:1151–210. 10.1016/S0140-6736(17)32152-9 28919116 PMC5605883

[2] Zhou M Wang H Zhu J Chen W Wang L Liu S Cause-specific mortality for 240 causes in china during 1990-2013: a systematic subnational analysis for the global burden of disease study 2013. *Lancet.* (2016) 387:251–72. 10.1016/S0140-6736(15)00551-6 26510778

[3] Liu S Li Y Zeng X Wang H Yin P Wang L Burden of cardiovascular diseases in China, 1990-2016: findings from the 2016 global burden of disease study. *JAMA Cardiol.* (2019) 4:342–52. 10.1001/jamacardio.2019.0295 30865215 PMC6484795

[4] Hopkins PN. Molecular biology of atherosclerosis. *Physiol Rev.* (2013) 93:1317–542. 10.1152/physrev.00004.2012 23899566

[5] Li J Huang L. [Role of platelets in the pathogenesis of atherothrombosis]. *Zhonghua Xin Xue Guan Bing Za Zhi.* (2014) 42:175–7. 24735635

[6] Jain K Tyagi T Patell K Xie Y Kadado AJ Lee SH Age associated non-linear regulation of redox homeostasis in the anucleate platelet: implications for Cvd Risk Patients. *EBioMedicine.* (2019) 44:28–40. 10.1016/j.ebiom.2019.05.022 31130473 PMC6604369

[7] Bertling A Fender AC Schungel L Rumpf M Mergemeier K Geissler G Reversibility of platelet P2y12 inhibition by platelet supplementation: *ex vivo* and *in vitro* comparisons of prasugrel, clopidogrel and ticagrelor. *J Thromb Haemost.* (2018) 16:1089–98. 10.1111/jth.14014 29582544

[8] Ornelas A Zacharias-Millward N Menter DG Davis JS Lichtenberger L Hawke D Beyond Cox-1: the effects of aspirin on platelet biology and potential mechanisms of chemoprevention. *Cancer Metastasis Rev.* (2017) 36:289–303. 10.1007/s10555-017-9675-z 28762014 PMC5557878

[9] Hao Q Tampi M O’Donnell M Foroutan F Siemieniuk RA Guyatt G. Clopidogrel plus aspirin versus aspirin alone for acute minor ischaemic stroke or high risk transient ischaemic attack: systematic review and meta-analysis. *BMJ.* (2018) 363:k5108. 10.1136/bmj.k5108 30563866 PMC6298178

[10] Zheng SL Roddick AJ. Association of aspirin use for primary prevention with cardiovascular events and bleeding events a systematic review and meta-analysis. *JAMA J Am Med Assoc.* (2019) 321:277–87. 10.1001/jama.2018.20578 30667501 PMC6439678

[11] Sesso HD Liu S Gaziano JM Buring JE. Dietary lycopene, tomato-based food products and cardiovascular disease in women. *J Nutr.* (2003) 133:2336–41. 10.1093/jn/133.7.2336 12840203

[12] de Lorgeril M Salen P Martin JL Monjaud I Delaye J Mamelle N. Mediterranean diet, traditional risk factors, and the rate of cardiovascular complications after myocardial infarction: final report of the lyon diet heart study. *Circulation.* (1999) 99:779–85. 10.1161/01.cir.99.6.779 9989963

[13] Hak AE Stampfer MJ Campos H Sesso HD Gaziano JM Willett W Plasma carotenoids and tocopherols and risk of myocardial infarction in a low-risk population of US male physicians. *Circulation.* (2003) 108:802–7. 10.1161/01.CIR.0000084546.82738.89 12900344

[14] O’Kennedy N Crosbie L van Lieshout M Broom JI Webb DJ Duttaroy AK. Effects of antiplatelet components of tomato extract on platelet function *in vitro* and ex vivo: a time-course cannulation study in healthy humans. *Am J Clin Nutr.* (2006) 84:570–9. 10.1093/ajcn/84.3.570 16960171

[15] O’Kennedy N Crosbie L Song HJ Zhang X Horgan G Duttaroy AKA. Randomised controlled trial comparing a dietary antiplatelet, the water-soluble tomato extract fruitflow, with 75 Mg aspirin in healthy subjects. *Eur J Clin Nutr.* (2017) 71:723–30. 10.1038/ejcn.2016.222 27876806 PMC5470100

[16] Lordan R Tsoupras A Zabetakis I. Platelet activation and prothrombotic mediators at the nexus of inflammation and atherosclerosis: potential role of antiplatelet agents. *Blood Rev.* (2021) 45:100694. 10.1016/j.blre.2020.100694 32340775

[17] O’Kennedy N Duss R Duttaroy AK. Dietary antiplatelets: a new perspective on the health benefits of the water-soluble tomato concentrate fruitflow^®^. *Nutrients.* (2021) 13:2184. 10.3390/nu13072184 34201950 PMC8308204

[18] Tourdot BE Conaway S Niisuke K Edelstein LC Bray PF Holinstat M. Mechanism of race-dependent platelet activation through the protease-activated receptor-4 and Gq signaling axis. *Arterioscler Thromb Vasc Biol.* (2014) 34:2644–50. 10.1161/ATVBAHA.114.304249 25278289 PMC4239175

[19] Infeld M Friede KA San TR Knickerbocker HJ Ginsburg GS Ortel TL Platelet reactivity in response to aspirin and ticagrelor in African-Americans and European-Americans. *J Thromb Thrombolysis.* (2021) 51:249–59. 10.1007/s11239-020-02327-w 33159252 PMC7889728

[20] Flammer AJ Sudano I Wolfrum M Thomas R Enseleit F Periat D Cardiovascular effects of flavanol-rich chocolate in patients with heart failure. *Eur Heart J.* (2012) 33:2172–80. 10.1093/eurheartj/ehr448 22173910

[21] Shi Y Williamson G. Quercetin lowers plasma uric acid in pre-hyperuricaemic males: a randomised, double-blinded, placebo-controlled, cross-over trial. *Br J Nutr.* (2016) 115:800–6. 10.1017/S0007114515005310 26785820

[22] Katada S Oishi S Yanagawa K Ishii S Oki M Matsui Y Concomitant use of tea catechins affects absorption and serum triglyceride-lowering effects of monoglucosyl hesperidin. *Food Funct.* (2021) 12:9339–46. 10.1039/d1fo01917a 34606551

[23] Cicero AFG Caliceti C Fogacci F Giovannini M Calabria D Colletti A Effect of apple polyphenols on vascular oxidative stress and endothelium function: a translational study. *Mol Nutr Food Res.* (2017) 61. *P, 10.1002/mnfr.201700373 28755406

[24] Zhang H Xu Z Zhao H Wang X Pang J Li Q Anthocyanin supplementation improves anti-oxidative and anti-inflammatory capacity in a dose-response manner in subjects with dyslipidemia. *Redox Biol.* (2020) 32:101474. 10.1016/j.redox.2020.101474 32179241 PMC7078384

[25] Xu ZL Xie JW Zhang HY Pang J Li Q Wang X Anthocyanin supplementation at different doses improves cholesterol efflux capacity in subjects with dyslipidemia-a randomized controlled trial. *Eur J Clin Nutr.* (2020) 75:345–54. 10.1038/s41430-020-0609-4 32317748

[26] Ya F Tian J Li Q Chen L Ren J Zhao Y Cyanidin-3-O-beta-glucoside, a natural polyphenol, exerts proapoptotic effects on activated platelets and enhances megakaryocytic proplatelet formation. *J Agric Food Chem.* (2018) 66:10712–20. 10.1021/acs.jafc.8b03266 30226049

[27] Patrono C Rocca B. Measurement of thromboxane biosynthesis in health and disease. *Front Pharmacol.* (2019) 10:1244. 10.3389/fphar.2019.01244 31736753 PMC6832017

[28] Ps A Rc A Ncr A Src B. Serum thromboxane B2 but not soluble P-Selectin levels identify ischemic stroke patients with persistent platelet reactivity while on aspirin therapy. *Thromb Res.* (2021) 208:92–8. 10.1016/j.thromres.2021.10.021 34742142

[29] Rossaint J Kuhne K Skupski J Van Aken H Looney MR Hidalgo A Directed transport of neutrophil-derived extracellular vesicles enables platelet-mediated innate immune response. *Nat Commun.* (2016) 7:13464. 10.1038/ncomms13464 27845343 PMC5116072

[30] Gogoi D Arora N Kalita B Sarma R Islam T Ghosh SS Anticoagulant mechanism, pharmacological activity, and assessment of preclinical safety of a novel fibrin(Ogen)olytic serine protease from leaves of *Leucas indica*. *Sci Rep.* (2018) 8:6210. 10.1038/s41598-018-24422-y 29670183 PMC5906637

[31] Barros T Batista DO Torrentes de Carvalho A Costa Faria NRD Barreto-Vieira DF Jacome FC Different aspects of platelet evaluation in dengue: measurement of circulating mediators, ability to interact with the virus, the degree of activation and quantification of intraplatelet protein content. *Virus Res.* (2019) 260:163–72. 10.1016/j.virusres.2018.09.013 30282001

[32] Yang Y Shi ZY Reheman A Jin JW Li CL Wang YM Plant food delphinidin-3-glucoside significantly inhibits platelet activation and thrombosis: novel protective roles against cardiovascular diseases. *PLoS One.* (2012) 7:e37323. 10.1371/journal.pone.0037323 22624015 PMC3356278

[33] Ya FL Xu XR Shi YL Gallant RC Song FL Zuo X Coenzyme Q10 upregulates platelet Camp/Pka pathway and attenuates integrin alpha Iib beta 3 signaling and thrombus growth. *Mol Nutr Food Res.* (2019) 63:1900662. 10.1002/mnfr.201900662 31512815

[34] Xu XR Wang YM Adili R Ju LN Spring CM Jin JW Apolipoprotein a-Iv binds Alpha Iib beta 3 integrin and inhibits thrombosis. *Nat Commun.* (2018) 9:3608. 10.1038/s41467-018-05806-0 30190457 PMC6127106

[35] O’Kennedy N Raederstorff D Duttaroy AK. Fruitflow^®^ : the first european food safety authority-approved natural cardio-protective functional ingredient. *Eur J Nutr.* (2017) 56:461–82. 10.1007/s00394-016-1265-2 27388464 PMC5334395

[36] O’Kennedy N Crosbie L Whelan S Luther V Horgan G Broom JI Effects of tomato extract on platelet function: a double-blinded crossover study in healthy humans. *Am J Clin Nutr.* (2006) 84:561–9. 10.1093/ajcn/84.3.561 16960170

[37] Nieswandt B Watson SP. Platelet-collagen interaction: is gpvi the central receptor? *Blood.* (2003) 102:449–61. 10.1182/blood-2002-12-3882 12649139

[38] Kaplan KL Owen J. Plasma levels of beta-thromboglobulin and platelet factor 4 as indices of platelet activation in vivo. *Blood.* (1981) 57:199–202. 10.1182/blood.v57.2.199.bloodjournal572199 6160890

[39] Die F Ze-zhong T Xi-lin M Xiao Z Fu-li Y Yan Y. Fruitflow, a water-soluble tomato concentrate, inhibits platelet activation, aggregation and thrombosis by regulating the signaling pathway of Pi3k/Akt and Mapks. *J Sun Yat-sen Univ. (Med. Sci.)* (2020) 41:243–50.

[40] Zhu Y Xia M Yang Y Liu F Li Z Hao Y Purified anthocyanin supplementation improves endothelial function *via* no-Cgmp activation in hypercholesterolemic individuals. *Clin Chem.* (2011) 57:1524–33. 10.1373/clinchem.2011.167361 21926181

[41] Song FL Zhu YN Shi ZY Tian JJ Deng XJ Ren J Plant food anthocyanins inhibit platelet granule secretion in hypercholesterolaemia: involving the signalling pathway of Pi3k-Akt. *Thromb Haemost.* (2014) 112:981–91. 10.1160/Th13-12-1002 25077916

[42] Naruszewicz M Laniewska I Millo B Dluzniewski M. Combination therapy of statin with flavonoids rich extract from chokeberry fruits enhanced reduction in cardiovascular risk markers in patients after myocardial infraction (Mi). *Atherosclerosis.* (2007) 194:e179–84. 10.1016/j.atherosclerosis.2006.12.032 17320090

[43] Soltani R Hakimi M Asgary S Ghanadian SM Keshvari M Sarrafzadegan N. Evaluation of the effects of *Vaccinium arctostaphylos* L. Fruit extract on serum lipids and Hs-Crp levels and oxidative stress in adult patients with hyperlipidemia: a randomized, double-blind, placebo-controlled clinical trial. *Evid Based Complement Alternat Med.* (2014) 2014:217451. 10.1155/2014/217451 24587807 PMC3920853

[44] Schror K. Aspirin and Platelets: the antiplatelet action of aspirin and its role in thrombosis treatment and prophylaxis. *Semin Thromb Hemost.* (1997) 23:349–56. 10.1055/s-2007-996108 9263351

[45] Rafferty M Walters MR Dawson J. Anti-platelet therapy and aspirin resistance–clinically and chemically relevant? *Curr Med Chem.* (2010) 17:4578–86. 10.2174/092986710794182962 21062249

[46] Uddin M Biswas D Ghosh A O’Kennedy N Duttaroy AK. Consumption of Fruitflow^®^ lowers blood pressure in pre-hypertensive males: a randomised, placebo controlled, double blind, cross-over study. *Int J Food Sci Nutr.* (2018) 69:494–502. 10.1080/09637486.2017.1376621 28918674

[47] Zhang S Chen H Li C Chen B Gong H Zhao Y Water-soluble tomato extract fruitflow alters the phosphoproteomic profile of collagen-stimulated platelets. *Front Pharmacol.* (2021) 12:746107. 10.3389/fphar.2021.746107 34646142 PMC8502824

[48] Aslam M Sedding D Koshty A Santoso S Schulz R Hamm C Nucleoside triphosphates inhibit Adp, Collagen, and epinephrine-induced platelet aggregation: role of P2y_1_ and P2y_12_ receptors. *Thromb Res.* (2013) 132:548–57. 10.1016/j.thromres.2013.08.021 24071464

[49] Bojic M Debeljak Z Medic-Saric M Tomicic M. Interference of selected flavonoid aglycons in platelet aggregation assays. *Clin Chem Lab Med.* (2012) 50:1403–8. 10.1515/cclm-2011-0960 22868805

[50] Vita JA. Polyphenols and cardiovascular disease: effects on endothelial and platelet function. *Am J Clin Nutr.* (2005) 81(Suppl. 1):292S–7S. 10.1093/ajcn/81.1.292S 15640493

[51] Ntzouvani A Antonopoulou S Fragopoulou E Kontogianni MD Nomikos T Mikellidi A Effect of differently fed farmed gilthead sea bream consumption on platelet aggregation and circulating haemostatic markers among apparently healthy adults: a double-blind randomized crossover trial. *Nutrients.* (2021) 13:286. 10.3390/nu13020286 33498445 PMC7909403

[52] Gavriil L Detopoulou M Petsini F Antonopoulou S Fragopoulou E. Consumption of plant extract supplement reduces platelet activating factor-induced platelet aggregation and increases platelet activating factor catabolism: a randomised, double-blind and placebo-controlled trial. *Br J Nutr.* (2019) 121:982–91. 10.1017/S0007114519000308 30940217

[53] Tian Z Li K Fan D Zhao Y Gao X Ma X Dose-dependent effects of anthocyanin supplementation on platelet function in subjects with Dyslipidemia: a randomized clinical trial. *EBioMedicine.* (2021) 70:103533. 10.1016/j.ebiom.2021.103533 34392146 PMC8374375

