# Supplementary material for: Four-Week Supplementation of Water-Soluble Tomato Extract Attenuates Platelet Function in Chinese Healthy Middle-Aged and Older Individuals: A Randomized, Double-Blinded, and Crossover Clinical Trial
Source: Front Nutr. 2022 Jun 1;9:891241. doi: 10.3389/fnut.2022.891241 (PMC9199899; doi:10.3389/fnut.2022.891241)
Supplement: Supplementary file 1 [file Table_1.docx]

Supplement Trial Protocol

Information described in this protocol formed part of the original submission.

Trial Protocol

Study Aims

The objective of this study was to determine the effect and safety of 4-week administration of WTE on platelet function in Chinese middle-aged and older individuals.

Study Hypotheses

Our hypothesis was that WTE supplementation for 4 weeks could moderately reduce platelet activation, aggregation and granule secretion in Chinese healthy middle-aged and older individuals, and these effects are safe.

Study Design
This was a randomized crossover-designed controlled trial. First, a total of 105 eligible subjects were stratified by gender and then randomly divided into two group 1 or group 2 by random numbers generated by SPSS v22.0 (SPSS Inc., Chicago, IL, USA).

Trial Protocol Approval
The Ethics Committee of Sun Yat-sen University approved the study protocol. All participants signed written informed consent prior to enrollment. Informed consent was obtained from all participants.

Trial and Protocol Registration
This trial was registered at Chinese clinical trial registry as ChiCTR-POR-17012927.

Study Population
The inclusion criteria were: (1) men or women aged 40 to 65 years; (2) without serious vascular or hematological diseases; (3) normal laboratory tests for hematuria, liver and kidney function, blood glucose and lipids, etc. The exclusion criteria were: (1) with the history of hypertension, infectious disease, hemostatic disorders, diabetes mellitus, cardiovascular diseases (CVDs); (2) agents use that could influence platelet function; (3) lactating or pregnant women; (4) allergic to tomatoes and their ingredients.

Recruitment Sources and Procedures
 Recruitment strategies included promotions at social networking, local senior/community centers and medical clinics, medical record review and clinicians’ recommendations. To reduce potential expectation bias, participants were informed that the study would be comparing two different groups and that they would be assigned to any group at random.

All the subjects were recruited from the medical examination center of the first affiliated hospital of Sun Yat-sen University and community health center through flyers, medical record reviews, or clinicians’ recommendations in Guangzhou, Guangdong, China. This study was approved by the Ethics Committee of Sun Yat-sen University (NO. 2016036), and was conducted in strict accordance with the principles of the Declaration of Helsinki.

Randomization and Blinding
Blinding was performed according to previous research^1,2^. Briefly, a technician who did not participate in the experiments, data collection or analysis was in charge of randomization and the management of the packaged supplements. Participants, investigators and laboratory technicians were blinded to the treatment assignments until the conclusion of the trial.

WTE tablets and placebo tablets were provided by DSM (Netherlands). The WTE tablets contain 150 mg WTE which comprise nucleosides, polyphenols and flavonoids purified from fresh tomatoes (Lycopersicon esculentum) by using solid-phase extraction, as described in previous studies^3^. The placebo tablets contained only maltodextrin. The weight, appearance, taste, and packaging of WTE tablets and placebo tablets were same.

Intervention

Individuals in group 1 took one WTE tablet (150 mg/d) and individuals in group 2 take one placebo tablet daily for 4 weeks, followed by a 2-week washout period. Then, the two groups switched groups and continued to take WTE (150 mg/d) or placebo tablets for another 4 weeks**.** During the 10-week trial period, all subjects were instructed to maintain their usual diet and lifestyle, but to refrain from tomatoes and tomato products. The 24-hour dietary recall data were recorded on 3 consecutive days, and international physical activity questionnaire (IPAQ) scores were collected via face-to-face interviews at baseline and after intervention

Data Collection and Outcome Measurements **Dietary habits and physical activity.** Participants were asked to maintain their usual dietary intake and physical activities to record their dietary habits and physical activity during the trial. The 24-hour dietary recall data on 3 consecutive days were collected at baseline and after intervention. Nutrient intakes were calculated using a computer-aided nutritional analysis program for professionals (Chinese Food Composition Table) as our previous studies^1,2^. Physical activity status was measured using the International Physical Activity Questionnaire (IPAQ), and outcome was presented as metabolic equivalent task (MET) hours per week (MET-h/wk)^4,5^.

Anthropometric analyses. Anthropometric measurements were performed by a trained examiner as previously described^1,2^. Body weight (BW), body height (BH), waist circumference (WC), and hip circumference (HC) were measured according to the standard protocols. Body mass index (BMI) was calculated based on BW and BH: BMI (kg/m^2^) = BW (kg)/BH^2^ (m^2^). The waist hip ratio (WHR) was calculated based on WC and HC: WHR (%) = WC (cm)/HC (cm) × 100%. Heart rate and blood pressure (BP), including systolic blood pressure (SBP) and diastolic blood pressure (DBP) were measured using a validated oscillometric technique (Omron U30 Intellisense, JPN). BP and heart rate were determined in the non- dominant upper arm after a 20-minute resting period on each arm twice. Four values were recorded at 2-minute intervals, and the average of these measures was considered. All measurements were obtained using standardized procedures and periodically calibrated instruments. Individual information was collected by the trained staff via face-to-face interview based on a structured questionnaire on socio-demographic data, dietary habits, and living habits.

Biological samples collection and assessment of biochemical biomarkers. Fasting blood samples were obtained at 8-9 AM from each subject’s antecubital vein with a 21-gauge needle at the beginning and at weeks 4, 6 and 10 of the trial. Whole human blood was collected into a Vacutainer tube containing 3.8% sodium citrate (1/9, v/v) via venipuncture. The blood sample was then centrifuged to obtain plasma or platelet-rich plasma (PRP)^6^. PRP was immediately used to assay platelet aggregation and activation or other related parameters. Plasma samples were stored at -80℃ until subsequent analyses.

Fasting blood samples were subjected to LDL-C, HDL-C, serum TG, serum TC, fasting blood glucose (FBG), urea, uric acid (UA), creatinine, alanine aminotransferase, total protein, albumin and globulin. Lipid profile analyses were performed on the Cobas c311 automated assay analyzer (c311, Roche Diagnostics, Switzerland). Enzymatic methods were used to determine the concentrations of HDL-C, LDL-C, TC, and TG. Other biochemical analyses included fasting blood glucose (FBG), urea, UA, creatinine, alanine aminotransferase, total protein, albumin and globulin were measured using the Cobas c311 automated assay analyzer. Prothrombin time (PT) activated partial thromboplastin time (APTT), thrombin clotting time (TT), and plasma fibrinogen (Fib) estimations were performed on a Sysmex5100 system (Siemens Healthineers, Malvern, USA).

The platelet granule contents including platelet factor 4 (PF4) (Abcam, UK), β-thromboglobulin (β-TG) (Abcam, UK), thromboxane A2 (TXA2) (R&D Systems, USA) in plasma were tested using an ELISA kit according to the manufacturer’s instructions.

**Flow cytometric analysis of platelet activation**. Analysis of platelet activation was tested via flow cytometer as previously described^7,8^. Freshly isolated PRP were incubated with different fluorescent-labeled antibodies activated GPⅡbⅢa (FITC-conjugated mouse anti-human PAC-1, BD Biosciences, San Jose, CA, USA), and P-selectin (FITC-conjugated mouse anti-human CD62p, BD Biosciences, San Jose, CA, USA) at room temperature for 30 min. Platelet activation was initiated by adding 100 µM adenosine diphosphate (ADP) ADP or 10µg/mL collagen at room temperature, followed by fixation with 1% paraformaldehyde (pH 7.2) before analysis. All samples were analyzed via a calibrated CytoFLEX flow cytometer (Beckman Coulter, CA, USA) ^8^.

Assessment of **platelet aggregation.** Platelet aggregation was performed by a Chronolog aggregometer (Chrono‐Log Corp., PA, USA) as previously described^8,9^.  Briefly, PRP prepared from subjects with dyslipidemia was stimulated by ADP or collagen at baseline and after intervention. Platelet aggregation was evaluated via a Chronolog aggregometer (Chrono‐Log Corp., PA, USA) in PRP (3.0 × 10^8^ platelets mL^−1^) at 37 °C with a sample stir speed of 1000 rpm, and the change in light transmission was monitored and recorded for at least 6 min.

Class Size
Use PASS software (version 11.0, NCSS Inc.) to estimate the sample size required for the study based on the conventional assumption of a two-tailed α level of 0.05 and β level of 0.10. According to the previous study^10^, the change rates of platelet aggregation in the intervention group and the control group were - 9.7% and - 3.1% after the intervention of WTE. Based on a two-tailed α level of 0.05 and β level of 0.10, 42 participants in each group were needed. Considering a 10% loss to follow-up rate, 47 subjects are needed in each group at least.

Drop-outs
Unavoidable dropouts, from causes such as death, onset of severe illness, or other medical complications, were anticipated. Based on our prior studies, we estimated a 10% dropout rate for the overall study. The proposed sample size took into account this anticipated dropout rate.

Program Fidelity
The investigators were trained by the unified investigation method, and the on-site investigation could be started after passing the examination. Before the formal follow-up survey, the reliability (repeatability) and validity (accuracy) of all survey tools, including the questionnaire, have been tested, and the formal survey can be conducted only after the allowable error of the relevant indicators of the major is met. For the laboratory test items, the quality control values of standard samples and samples of survey population were set, and the quality control samples were determined at the same time when each batch of samples were tested.

Statistical Analysis
All statistical analyses were two-tailed and performed using SPSS v22.0 (SPSS Inc., Chicago, IL, USA). Statistical significance was defined as *P*< 0.05.

**Descriptive statistics.** SPSS 22.0 software was used for statistical analysis. In the clinical study, the data are expressed as the mean ± standard error of the mean (SEM) unless otherwise stated. For categorical variables, analysis was performed using the chi-square test. The changes after 4-week WTE supplementation or placebo supplementation were calculated as values after 4-week intervention (at 4 weeks or 10 weeks) deducted from the values at baseline (at 0 week or after 2-week washout period). For continuous variables, variables were checked for normal distribution and were assessed by Kruskal-Wallis test if they were not normally distributed.

**Statistical analysis.** The changes after intervention were calculated as values after 4-week intervention deducted from the values at baseline. The comparability of the two groups at baseline were assessed by one-way analysis of variance (ANOVA). The effects of treatments were evaluated using one-way ANOVA, comparisons of each index at baseline (at 0 week or after 2-week washout period) and after 4-week intervention (at 4 weeks or 10 weeks) between two groups were conducted using the Student’s *t*-test for unpaired data. The mean changes of each index between two groups after 4-weeks intervention were compared using the Student’s t-test for unpaired data. To explore the reversibility of 4-week supplementation of WTE, one-way ANOVA were used to assess changes between two groups at baseline, 4weeks, 6 weeks and 10 weeks. Differences were considered statistically significant at P < 0.05.

**References:**

1. Xu ZL, Xie JW, Zhang HY, et al. Anthocyanin supplementation at different doses improves cholesterol efflux capacity in subjects with dyslipidemia-a randomized controlled trial. *Eur J Clin Nutr.* 2020.

2. Zhang H, Xu Z, Zhao H, et al. Anthocyanin supplementation improves anti-oxidative and anti-inflammatory capacity in a dose-response manner in subjects with dyslipidemia. *Redox Biol.* 2020;32:101474.

3. O'Kennedy N, Crosbie L, van Lieshout M, Broom JI, Webb DJ, Duttaroy AK. Effects of antiplatelet components of tomato extract on platelet function in vitro and ex vivo: a time-course cannulation study in healthy humans. *The American journal of clinical nutrition.* 2006;84(3):570-579.

4. Hagstromer M, Oja P, Sjostrom M. The International Physical Activity Questionnaire (IPAQ): a study of concurrent and construct validity. *Public Health Nutr.* 2006;9(6):755-762.

5. Craig CL, Marshall AL, Sjostrom M, et al. International physical activity questionnaire: 12-country reliability and validity. *Med Sci Sports Exerc.* 2003;35(8):1381-1395.

6. Ya F, Tian J, Li Q, et al. Cyanidin-3-O-beta-glucoside, a Natural Polyphenol, Exerts Proapoptotic Effects on Activated Platelets and Enhances Megakaryocytic Proplatelet Formation. *J Agric Food Chem.* 2018;66(41):10712-10720.

7. Yang Y, Shi ZY, Reheman A, et al. Plant Food Delphinidin-3-Glucoside Significantly Inhibits Platelet Activation and Thrombosis: Novel Protective Roles against Cardiovascular Diseases. *PloS one.* 2012;7(5).

8. Ya FL, Xu XR, Shi YL, et al. Coenzyme Q10 Upregulates Platelet cAMP/PKA Pathway and Attenuates Integrin alpha IIb beta 3 Signaling and Thrombus Growth. *Molecular Nutrition & Food Research.* 2019;63(23).

9. Xu XR, Wang YM, Adili R, et al. Apolipoprotein A-IV binds alpha IIb beta 3 integrin and inhibits thrombosis. *Nature Communications.* 2018;9:3608.

10. O'Kennedy N, Crosbie L, Song HJ, Zhang X, Horgan G, Duttaroy AK. A randomised controlled trial comparing a dietary antiplatelet, the water-soluble tomato extract Fruitflow, with 75 mg aspirin in healthy subjects. *Eur J Clin Nutr.* 2017;71(6):723-730.
