# Supplementary material for: Four-Week Supplementation of Water-Soluble Tomato Extract Attenuates Platelet Function in Chinese Healthy Middle-Aged and Older Individuals: A Randomized, Double-Blinded, and Crossover Clinical Trial
Source: Front Nutr. 2022 Jun 1;9:891241. doi: 10.3389/fnut.2022.891241 (PMC9199899; doi:10.3389/fnut.2022.891241)
Supplement: Supplementary file 2 [file Table_2.docx]

**Supplemental Table 1 The composition of water-soluble tomato extract.**

**Supplemental Table 2 Routine blood tests of all participants at baseline and after treatment.**

**Supplemental Table 3 Daily dietary intakes and physical activities at baseline and after treatment.**

**Supplemental Table 4 Changes of platelet activation and aggregation after 4-week treatment stratified by gender.**

**Supplemental Table 5 Changes of platelet granule secretion after 4-week treatment stratified by gender.**

**Supplemental Table 6 Platelet activation and aggregation of all participants at baseline and after treatment.**

**Supplemental Table 7 Platelet granule secretion of all participants at baseline and after treatment.**

**Supplemental Table 1 The composition of water-soluble tomato extract.**

| **Components** | **Content** |
| --- | --- |
| **Physicochemical parameters** |  |
| Moisture content (%) | 3.3 |
| Ash content (%) | 1.9 |
| **Microbe Index** |  |
| Total number of colonies (CFU/g) | ≤1000 |
| Mold and yeast (CFU/g) | ≤100 |
| Escherichia coli | - |
| Salmonella | - |
| Staphylococcus aureus | - |
| Pseudomonas aeruginosa | - |
| **Active ingredient** |  |
| nucleosides, polyphenols and flavonoids (mg/g) | 23 |

**Supplemental Table 2 Routine blood tests of all participants at baseline and after treatment.**

|  | **Group 1（n=54）** | | **Group 2（n=51）** | |
| --- | --- | --- | --- | --- |
|  | **baseline** | **after intervention** | **baseline** | **after intervention** |
| WBC (10^^9^/L) | 5.5±1.4 | 5.6±1.2 | 5.9±1.4 | 6.1±1.7 |
| RBC (10^^9^/L) | 4.5±0.4 | 4.5±0.4 | 4.6±0.4 | 4.5±0.4 |
| HGB (g/L) | 133.0±14.2 | 132.7±13.3 | 137.2±14.8 | 135.6±15.2 |
| HCT (%) | 40.3±3.6 | 40.0±3.4 | 41.6±4.1 | 40.8±4.0 |
| MCV (fl) | 90.5±6.7 | 90.0±6.6 | 91.2±6.0 | 90.8±6.0 |
| MCH (pg) | 29.9±2.7 | 29.9±2.6 | 30.1±2.4 | 30.2±2.6 |
| MCHC (g/L) | 329.5±10.6 | 331.4±10.3 | 329.6±9.3 | 332.1±11.8 |
| RDW (%) | 13.5±1.6 | 13.4±1.2 | 13.4±1.6 | 13.3±1.2 |
| PLT (10^^9^/L) | 226.7±58.9 | 238.2±62.4 | 234.4±53.0 | 246.7±55.1 |
| MPV (fl) | 9.9±0.9 | 10.0±1.0 | 9.5±1.2 | 9.6±1.1 |
| PDW (%) | 16.4±0.6 | 16.3±0.5 | 16.3±1.1 | 16.3±0.5 |
| LYM (10^^9^/L) | 2.0±0.6 | 1.8±0.5 | 2.0±0.6 | 2.0±0.6 |
| MON (10^^9^/L) | 0.4±0.1 | 0.4±0.1 | 0.4±0.2 | 0.4±0.1 |
| NEU (10^^9^/L) | 3.0±1.0 | 3.3±1.0 | 3.5±1.2 | 3.6±1.3 |
| EOS (10^^9^/L) | 0.1±0.1 | 0.2±0.1 | 0.2±0.1 | 0.2±0.2 |
| BAS (10^^9^/L) | 0.04±0.02 | 0.04±0.02 | 0.05±0.03 | 0.04±0.02 |

The data are expressed as mean ± SEM. There were no significant differences for any variable between the two groups at the baseline or after intervention. WBC, white blood cell count; RBC, red blood cell count; HGB, hemoglobin; HCT, Hematocrit; MCV, mean corpuscular volume; MCH, mean corpuscular hemoglobin; MCHC, mean corpuscular hemoglobin contentration; RDW, red blood cell distribution width; PLT, platelet; MPV, mean platelet volume; PDW, platelet distribution width; LYM, lymphocyte; MON, monocyte; NEU, neutrophils; EOS, eosinophils; BAS, basophil.

**Supplemental Table 3 Daily dietary intakes and physical activities at baseline and after treatment.**

|  | **Group 1（n=54）** | | **Group 2（n=51）** | |
| --- | --- | --- | --- | --- |
|  | **baseline** | **after intervention** | **baseline** | **after intervention** |
| Total energy (kcal/d) | 1621.0±104.5 | 1533.1±70.6 | 1590.6±86.2 | 1486.5±70.6 |
| Total protein (g/d) | 72.8±4.2 | 69.0±3.1 | 73.0±4.3 | 67.1±3.3 |
| Carbohydrates (g/d) | 133.0±14.2 | 132.7±13.3 | 137.2±14.8 | 135.6±15.2 |
| Total lipids(g/d) | 40.3±3.6 | 40.0±3.4 | 41.6±4.1 | 40.8±4.0 |
| Cholesterol (mg/d) | 90.5±6.7 | 90.0±6.6 | 91.2±6.0 | 90.8±6.0 |
| Dietary fiber (g/d) | 29.9±2.7 | 29.9±2.6 | 30.1±2.4 | 30.2±2.6 |
| Vitamin C (mg/d) | 329.5±10.6 | 331.4±10.3 | 329.6±9.3 | 332.1±11.8 |
| Vitamin A (μg retinol equivalent/d) | 13.5±1.6 | 13.4±1.2 | 13.4±1.6 | 13.3±1.2 |
| Vitamin E (mg/d) | 226.7±58.9 | 238.2±62.4 | 234.4±53.0 | 246.7±55.1 |
| Physical activities (MET-h/week) | 9.9±0.9 | 10.0±1.0 | 9.5±1.2 | 9.6±1.1 |

The data are expressed as mean ± SEM. There were no significant differences for any variable between the two groups at the baseline or after intervention.

**Supplemental Table 4 Changes of platelet activation and aggregation after 4-week treatment stratified by gender**

|  | Male(N=62) | | | | Female(N=148) | | |
| --- | --- | --- | --- | --- | --- | --- | --- |
|  | Placebo  （n=31） | WTE  （n=31） | | *P****_ANOVA_^a^*** | Placebo  （n=74） | WTE  （n=74） | *P****_ANOVA_*** |
| ADP-induced platelet aggregation (%) | -0.2±3.9 | | -11.1±3.8 | 0.049 | -2.2±2.6 | -10.7±2.0 | 0.009 |
| collagen-induced platelet aggregation (%) | 4.1±2 | | -6.2±2.7 | 0.004 | 0.4±1.4 | -3.0±1.7 | 0.132 |
| ADP-induced platelet P-selectin (%) | 6.1±3.1 | | -5.6±2.5 | 0.004 | 4.3±1.6 | -7.5±1.9 | <0.001 |
| collagen-induced platelet P-selectin (%) | -1.7±2.6 | | -3.2±2.4 | 0.683 | -0.5±1.3 | -8.0±1.5 | <0.001 |
| ADP-induced activated platelet GPⅡbⅢa (%) | 8.7±3.7 | | -4.2±3.2 | 0.011 | 3.6±1.9 | -7.1±2.5 | 0.001 |
| collagen-induced activated platelet GPⅡbⅢa (%) | 5.1±2.6 | | -1.7±4.3 | 0.176 | 2.1±2.5 | -4.7±2.3 | 0.046 |

The data are expressed as mean ± SEM.

***^a^*:** One-way ANOVA were used to assess changes between two groups.

**Supplemental Table 5 Changes of platelet granule secretion after 4-week treatment stratified by gender**

|  | Male(N=62) | | | | Female(N=) | | |
| --- | --- | --- | --- | --- | --- | --- | --- |
|  | Placebo  （n=31） | WTE  （n=31） | | *P****_ANOVA_ ^a^*** | Placebo  （n=） | WTE  （n=） | *P****_ANOVA_*** |
| PF4 (ng/mL) | 47.2±61.7 | | -137.6±52.4 | 0.026 | 32.7±51.2 | -113.5±41.9 | 0.029 |
| β-TG (ng/mL) | -83.2±45.5 | | -99.8±46.9 | 0.801 | -53.8±29.3 | -142.2±33.8 | 0.05 |
| TXB_2_(ng/ml） | -2.5±4.7 | | -49.3±7.2 | <0.001 | -0.4±2.9 | -39.0±4.8 | <0.001 |

The data are expressed as mean ± SEM.

***^a^*:** One-way ANOVA were used to assess changes between two groups.

**Supplemental Table 6 Platelet activation and aggregation of all participants at baseline and after treatment.**

|  | **0 weeks** | | | **4 weeks** | | | **6 weeks** | | |
| --- | --- | --- | --- | --- | --- | --- | --- | --- | --- |
|  | **Group1** | **Group2** | **P**  **value** | **Group1** | **Group2** | **P**  **value** | **Group2** | **Group1** | **P**  **value** |
|  | **(n=54)** | **(n=51)** |  | **(n=54)** | **(n=51)** |  | **(n=51)** | **(n=54)** |  |
| ADP-induced platelet aggregation (%) | 68.2 (17.3) | 70.4 (13.8) | 0.475 | 62.1 (18.1) | 54.5 (16.5)^##^ | 0.028 | 75.3 (13.3) | 73.3 (14.1) | 0.448 |
| collagen-induced platelet aggregation (%) | 80.2 (10.3) | 81.0 (10.1) | 0.713 | 80.5 (9.00) | 76.5 (10.2)^#^ | 0.033 | 80.6 (7.14) | 82.4 (6.98) | 0.204 |
| ADP-induced platelet P-selectin (%) | 49.2 (11.3) | 52.4 (9.25) | 0.107 | 53.7 (14.4) | 45.3 (10.8)^##^ | 0.001 | 48.6 (10.8) | 50.4 (11.3) | 0.410 |
| ADP-induced activated platelet GPⅡbⅢa (%) | 44.3 (16.5) | 50.2 (17.8) | 0.082 | 49.6 (20.7) | 41.3 (22.0)^#^ | 0.049 | 54.6 (11.6) | 54.9 (9.55) | 0.884 |
| collagen-induced platelet P-selectin (%) | 30.4 (9.26) | 33.6 (10.7) | 0.106 | 33.0 (11.0) | 29.2 (11.1)^#^ | 0.082 | 32.0 (9.32) | 30.7 (9.32) | 0.461 |
| collagen-induced activated platelet GPⅡbⅢa (%) | 15.6 (9.13) | 16.3 (8.99) | 0.689 | 23.6 (12.1) | 16.7 (13.7) | 0.007 | 27.7 (16.9) | 26.1 (19.0) | 0.653 |

The data are expressed as mean ± SEM. ^#^P < 0.05, ^##^ P < 0.01, 0 weeks vs 4 weeks, assessed by unpaired Student’s t-test.

**Supplemental Table 7 Platelet granule secretion of all participants at baseline and after treatment.**

|  | **0 weeks** | | | **4 weeks** | | | **6 weeks** | | |
| --- | --- | --- | --- | --- | --- | --- | --- | --- | --- |
|  | **Group1** | **Group2** | **P**  **value** | **Group2** | **Group1** | **P**  **value** | **Group1** | **Group2** | **P**  **value** |
|  | **(n=54)** | **(n=51)** |  | **(n=54)** | **(n=51)** |  | **(n=51)** | **(n=54)** |  |
| β-TG (ng/mL) | 564 (209) | 521 (213) | 0.297 | 429 (194) | 421 (220)^#^ | 0.851 | 472 (139) | 472 (177) | 0.999 |
| PF4 (ng/mL) | 162 (47.4) | 151 (51.6) | 0.256 | 152 (58.7) | 105 (77.8)^##^ | 0.001 | 169 (33.3) | 169 (39.6) | 0.970 |
| TXA2(ng/ml） | 508 (355) | 392 (259) | 0.057 | 384 (213) | 217 (196)^##^ | <0.001 | 271 (170) | 402 (245) | 0.002 |

The data are expressed as mean ± SEM. ^#^P < 0.05, ^##^ P < 0.01, 0 weeks vs 4 weeks, assessed by unpaired Student’s t-test.
